# Accuracy and Clinical Safety of the FreeStyle Libre 2 System in an Internal Medicine Intermediate Care Unit

**DOI:** 10.7759/cureus.102150

**Published:** 2026-01-23

**Authors:** Helena Urbano Ferreira, Patrícia Ferreira, Catarina Faustino, Rui Ribeiro, Joana Queirós, Sandra Belo, Fernando Friões

**Affiliations:** 1 Department of Endocrinology, Diabetes and Metabolism, Unidade Local de Saúde de São João, Porto, PRT; 2 Faculty of Medicine, University of Porto, Porto, PRT; 3 Department of Internal Medicine, Unidade Local de Saúde de São João, Porto, PRT

**Keywords:** continuous glucose monitoring, critical care, hyperglycemia, hypoglycemia, point-of-care testing

## Abstract

Introduction: Hyperglycemia and glycemic variability in hospitalized patients are strongly associated with adverse outcomes, including increased mortality and infection rates. While continuous glucose monitoring (CGM) offers the potential for real-time surveillance, its accuracy in critical patient settings remains under scrutiny. This study evaluates the real-world accuracy and clinical safety of the FreeStyle Libre 2 system in an Internal Medicine Intermediate Care Unit (IMICU).

Methods: We conducted a prospective observational study of adult patients admitted to the IMICU at Unidade Local de Saúde de São João, located in Porto, Portugal. Participants utilized the FreeStyle Libre 2 system, and sensor glucose values were paired with reference capillary point-of-care (POC) measurements. Accuracy was assessed using the mean absolute relative difference (MARD) and point accuracy thresholds (percentage of readings within 15%/15 mg/dL, 20%/20 mg/dL, and 30%/30 mg/dL of reference). Clinical safety was evaluated using the Parkes (Consensus) Error Grid Analysis (EGA). Subgroup analyses were performed to assess performance across glycemic ranges, diabetes status, and clinical comorbidities.

Results: Twenty-three patients (mean age 72.39 ± 15.51 years; 14 (60.9%) male) were included, providing 414 paired glucose readings. The overall MARD was 18.62 ± 11.87%. On the Parkes Error Grid, 226 (99.03%) paired readings fell within Zones A and B, with one (0.24%) in Zone E. Point accuracy analysis showed 46.14% of readings within 15%/15 mg/dL, 61.11% within 20%/20 mg/dL, and 85.75% within 30%/30 mg/dL. MARD values did not differ significantly across hypoglycemic, euglycemic, and hyperglycemic ranges (p = 0.843) or between patients with and without diabetes (p = 0.401). Subgroup analysis revealed lower MARD in patients receiving intravenous fluid therapy (p < 0.001) and who presented with shock (p < 0.001).

Conclusion: Despite a higher numerical MARD typical of comparisons against POC reference methods, the FreeStyle Libre 2 system demonstrated a high degree of clinical safety (99.03% in acceptable zones) in the IMICU. Its consistent performance in both diabetic and non-diabetic cohorts supports its potential as an adjunctive surveillance tool for detecting glycemic trends in critical patient settings.

## Introduction

Hyperglycemia and marked glycemic variability are common during hospitalization and are strongly associated with adverse clinical outcomes, including increased risk of infection, prolonged length of stay, and higher mortality [1,2]. These risks are particularly pronounced in patients admitted to intermediate- and critical patient units, where acute illness, complex therapeutic regimens, and rapid metabolic shifts make glycemic control especially challenging [1].

Traditional intermittent point-of-care (POC) capillary glucose testing, although the standard of care in hospitalized patients, has important limitations: it may fail to detect rapid glycemic excursions, transient hypoglycemia, or postprandial hyperglycemia, potentially delaying therapeutic interventions [3]. Continuous glucose monitoring (CGM) has emerged as a promising tool to address these gaps, providing real-time glucose trends, alarms for hypo- or hyperglycemia, and a more comprehensive assessment of glycemic variability [3,4].

Recent studies have generally demonstrated good accuracy of CGM in hospitalized non-critical patients both with and without diabetes, with acceptable mean absolute relative difference (MARD) values and high concordance on the Clarke Error Grid [5-9]. However, the performance of CGM in critically ill or hemodynamically unstable patients remains uncertain. Conditions such as anemia, renal dysfunction, edema, fluid shifts, and hypoperfusion can negatively affect sensor accuracy [8,10], underscoring the need for cautious interpretation of CGM readings in complex clinical settings and reinforcing the continued role of POC glucose testing. In fact, current ADA consensus statements support the use of CGM in selected hospitalized patients, while recommending that POC glucose testing should continue to be performed alongside CGM for clinical decision-making [11].

While recent consensus suggests that CGM systems can be used safely in non-critical care settings and may be considered in select critical care scenarios with confirmatory POC testing, data regarding the "real-world" performance of these devices remains variable. For instance, Finn et al. (2023) demonstrated acceptable MARD values in critical and non-critical settings [5], yet other literature suggests that accuracy may degrade in specific subpopulations, such as those with severe edema or on vasopressors [12]. This variability underscores the importance of robust, real-world validation in specific clinical environments, particularly those units that bridge the gap between non-critical and critical care.

The aim of this exploratory, hypothesis-generating study was to assess the real-world performance and safety of the FreeStyle Libre 2 system in patients admitted to an Internal Medicine Intermediate Care Unit (IMICU) by comparing CGM readings with capillary blood glucose measurements and evaluating glycemic trends under routine clinical conditions.

## Materials and methods

Study design and setting

We conducted a prospective, single-center, observational study using a consecutive (serial) sampling technique at the IMICU of Unidade Local de Saúde de São João, a tertiary care hospital located in Porto, Portugal, between January and November 2025. The IMICU is a level 2 critical care unit providing continuous monitoring and non-invasive respiratory support, bridging the gap between the general ward and the intensive care unit (ICU). Adult patients (age ≥18 years) admitted to the unit were eligible for inclusion if they had a clinical indication for capillary glucose monitoring. Patients were included regardless of a prior history of diabetes mellitus and regardless of current systemic corticosteroid use. Indications for glucose monitoring included pre-existing diabetes mellitus, newly diagnosed diabetes during admission, anticipated or ongoing systemic corticosteroid therapy,* nil per os* (NPO; nothing by mouth) status, or administration of enteral or parenteral nutrition. Only patients capable of providing informed consent were included. Patients were excluded from the analysis if they had less than 12 hours of sensor data or fewer than four matched glucose pairs, or experienced sensor malfunction or data loss. A formal sample size calculation was not performed for this exploratory observational study. The sample size was determined by the availability of eligible patients admitted to the IMICU during the study period who met the inclusion criteria. Shock was defined clinically by the treating team as hemodynamic instability requiring aggressive intravenous fluid resuscitation, in accordance with routine IMICU practice.

Data collection

The FreeStyle Libre 2 sensor was placed on eligible patients at the initiation of glucose monitoring. The device was affixed to the posterior aspect of the upper arm, following manufacturer guidelines. Glucose measurements, which served as the reference standard, were obtained from standard fingerstick capillary samples using the FreeStyle Precision point-of-care glucose meter at clinically indicated time points. These measurements were usually performed before meals and at bedtime, with additional measurements performed as required according to clinical status. POC glucose was used as the reference standard because it guided all insulin dosing and clinical decisions in the IMICU. While this may not fully reflect plasma glucose values, it represents the clinically relevant comparator for evaluating CGM performance in this setting. Concurrent interstitial glucose readings were obtained from the sensor. All insulin dosing decisions were based solely on capillary glucose results. Clinical and demographic data were collected from medical records. Any POC measurements that returned an error code, specifically "HI" or "LO" readings, were excluded from the analysis. 

Statistical analysis

We evaluated the accuracy of the FreeStyle Libre 2 system using the paired capillary and interstitial glucose measurements. Global performance was assessed by the MARD and the Parkes (Consensus) Error Grid Analysis (EGA) [13]. In addition, point accuracy was determined by calculating the percentage of paired measurements that met standardized agreement criteria. For each pair, the tolerance for acceptable error was based on the greater of a fixed absolute value (in mg/dL) or a relative percentage of the capillary glucose value. This approach effectively sets 100 mg/dL as the critical threshold: an absolute error limit is applied at or below this value, and a percentage error limit is applied above it. We calculated agreement rates for three key limits: 15% or 15 mg/dL, 20% or 20 mg/dL, and 30% or 30 mg/dL [14].

Subgroup differences in MARD were tested using the Mann-Whitney U test. Intra- and inter-individual variability were calculated using the standard deviation of the relative differences to characterize sensor consistency. Early sensor performance (defined as measurements in the first 24 hours of sensor use) was assessed for correlation with the overall accuracy of the remaining data points using Pearson's correlation coefficient. Continuous variables are presented as mean ± SD, and categorical variables are presented as counts and percentages. All analyses were performed using IBM SPSS Statistics for Windows, version 29.0 (released 2022, IBM Corp., Armonk, NY), with a p-value less than 0.05 considered statistically significant.

Ethics statement

This study was approved by the Unidade Local de São João Ethics Committee (approval number 400/2024). All procedures were conducted in accordance with the ethical standards of the institutional and/or national research committee and with the 1964 Helsinki Declaration and its later amendments. Written informed consent was obtained from all individual participants included in the study.

## Results

Study population and data characteristics 

A total of 23 patients were included in the analysis (Table 1). The mean age was 72.39 ± 15.51 years, and the mean BMI was 27.8 ± 3.8 kg/m². There were 14 (60.9%) male and nine (39.1%) female patients. Fourteen (60.9%) patients had a history of diabetes, while nine (39.1%) did not. Comorbidities included anemia (17 (73.9%)), acidosis (9 (39.1%)), hypotension (7 (30.4%)), acute kidney injury (7 (30.4%)), and chronic kidney disease (7 (30.4%)). Glucocorticoids were administered to six (26.1%) patients. No patients had generalized edema or were receiving vasopressors at the time of monitoring. The mean duration of CGM use was 5.23 ± 6.14 days. A total of 414 paired CGM-POC glucose readings were obtained for analysis, with a median of 13.0 pairs per patient (IQR 5.5-26.5).

**Table 1:** Participant’s characteristics

Demographics and anthropometrics	
Age, years - mean ± SD	72.39 ± 15.51
Body mass index, kg/m^2^ - mean ± SD	27.8 ± 3.8
Sex - n (%)	
Male	14 (60.1)
Female	9 (39.9)
Diabetes status - n (%)	
History of diabetes	14 (60.1)
No history of diabetes	9 (39.9)
New-onset diabetes	0 (0.0)
Comorbidities and clinical status - n (%)	
Chronic kidney disease	7 (30.4)
Acute kidney injury	7 (30.4)
Anemia	17 (73.9)
Acidosis	9 (39.1)
Hypotension	7 (30.4)
Shock	2 (8.7)
Generalized edema	0 (0.0)
Drugs used during admission - n (%)	
Vasopressors	0 (0.0%)
Fluids	4 (17.4%)
Glucocorticoids	6 (26.1%)
Primary indication for monitoring - n (%)	
Diabetes mellitus	14 (60.9%)
Glucocorticoids	5 (21.7%)
Enteric nutrition	3 (13.0%)
Hypoglycemia in a patient without diabetes	1 (4.3%)
FreeStyle Libre 2 system use	
Duration, days - mean ± SD	5.23 ± 6.14
Pairs of readings per patient - median ± IQR	13.0 (5.5-26.5)

Accuracy and clinical safety 

The overall analytical performance of the FreeStyle Libre 2 system yielded an MARD of 18.62 ± 11.87% (Table 4). In terms of specific point agreement, 46.14% of paired readings were within 15% or 15 mg/dL of the reference measurement. This agreement rate increased to 61.11% of pairs falling within 20% or 20 mg/dL, and further to 85.75% of pairs within 30% or 30 mg/dL (Table 2). Regarding sensor consistency, the study yielded an intra-individual variability of 10.91% and an inter-individual variability of 6.40%.

**Table 2:** Point accuracy of the paired measurements

Point accuracy	Result
15% / 15 mg/dL	46.14%
20% / 20 mg/dL	61.11%
30% / 30 mg/dL	85.75%

**Figure 1:**
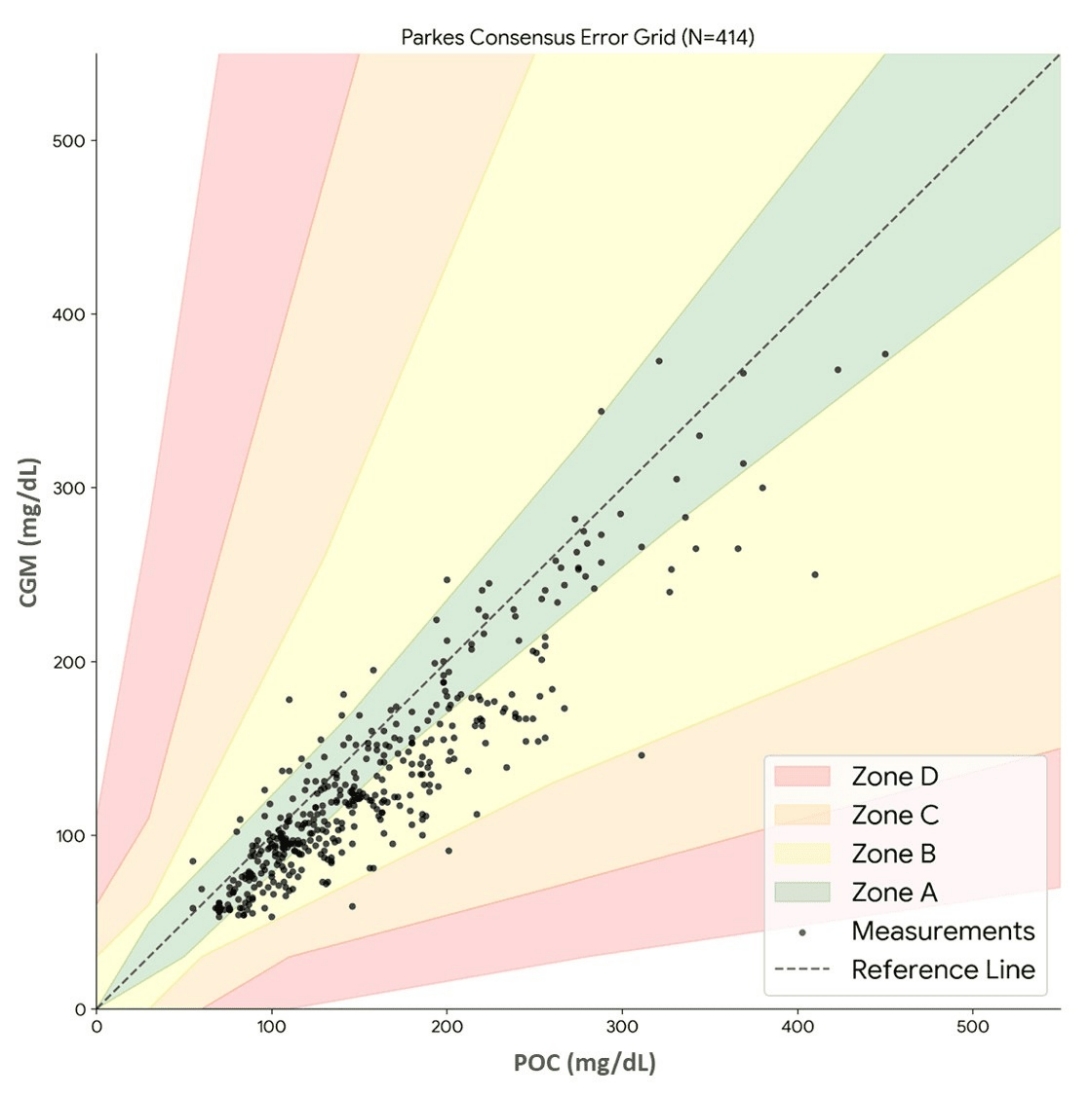
Parkes error grid analysis Image created by the authors with Python (version 3.11) and the Matplotlib library

Clinical safety was further assessed using the Parkes EGA (Figure 1, Table 3). The vast majority of paired readings fell within the clinically acceptable zones, with 226 (54.59%) located in Zone A (clinically accurate) and 184 (44.44%) in Zone B (benign errors). Consequently, 410 (99.03%) of all readings were considered clinically acceptable. Deviations outside these zones were rare (4 (0.97%)).

**Table 3:** Frequencies by zones of the Parkes (Consensus) Error Grid Analysis POC: point of care; CGM: continuous glucose monitoring

Zone	Percentage of pairs	Number of pairs	POC, mg/dL (mean ± SD)	CGM, mg/dL (mean ± SD)
A	54.59%	226	145.87 ± 70.65	135.26 ± 68.00
B	44.44%	184	167.86 ± 61.75	126.36 ± 50.68
C	0.72%	3	219.33 ± 84.01	98.67± 44.00
D	0.00%	0	N/A	N/A
E	0.24%	1	194.00	224.00

Subgroup analysis

Subgroup analysis results are presented in Table 4 and should be interpreted as exploratory and hypothesis-generating. Patients who received fluid therapy (13.79 ± 11.41 vs. 17.43 ± 11.28) and those with acute kidney injury (7 (30.43%); 17.47 ± 13.10% vs. 19.46 ± 11.24%) showed lower MARD values compared with their respective counterparts. In addition, patients presenting with shock (2 (8.7%)) showed numerically lower MARD values compared with their counterparts. No statistically significant differences in sensor accuracy were observed across other clinical variables, including age, body mass index categories, or the presence of diabetes, anemia, chronic kidney disease, or hypotension. Furthermore, performance remained consistent across different glycemic ranges, and sensor calibration status did not significantly impact accuracy, with comparable MARD values observed during the warm-up period versus the post-warm-up phase.

**Table 4:** Subgroup comparison of MARD MARD: mean absolute relative difference; SD: standard deviation. *Comparison of MARD between independent subgroups was performed using the Mann-Whitney U test.

Subgroup	Patients (n)	Pairs (n)	MARD ± SD (%)		U-value*	p-value*
Overall	23	414	18.62 ± 11.87		-	-
Glucose range						
Hypoglycemia (<70 mg/dL)	N/A	6	20.26 ± 17.27		851.0	0.843
Euglycemia (70–180 mg/dL)		287	18.33 ± 11.50		-	
Hyperglycemia (>180 mg/dL)		121	19.23 ± 12.50		17,989.5	
Calibration phase						
During warm-up period	20	57	17.36 ± 12.69		8,490.5	0.216
Post warm-up period		332	19.05 ± 11.76			
Age						
≥70 years	15	216	18.05 ± 12.13		10,299.5	0.146
<70 years	8	198	19.23 ± 11.57			
Body mass index						
<25	4	52	18.37 ± 12.17		-	0.280
25–30	6	100	15.56 ± 10.86		1,393	
≥30	2	35	16.45 ± 8.95		3,086	
Diabetes mellitus						
Yes	14	179	13.48 ± 5.15		19,302.5	0.401
No	9	235	12.44 ± 6.94			
Chronic kidney disease						
Yes	7	33	19.17 ± 13.62		7,592	0.950
No	16	356	18.77 ± 11.75			
Acute kidney injury						
Yes	7	128	17.47 ± 13.10		16,199.0	0.041
No	12	261	19.46 ± 11.24			
Anemia						
Yes	17	294	19.14 ± 12.68		17,808.5	0.681
No	6	95	17.77 ± 9.04			
Shock						
Yes	2	61	13.09 ± 8.81		-	-
No	21	328	19.86 ± 12.11			
Hypotension						
Yes	7	214	19.13 ± 11.45		22,977.5	0.359
No	16	175	18.40 ± 12.45			
Fluid therapy						
Yes	11	214	13.79 ± 11.41		10,942.0	<0.001
No	12	175	17.43 ± 11.28			

## Discussion

This study evaluated the accuracy of the FreeStyle Libre 2 system against capillary glucose measurements in patients admitted to an IMICU. The overall real-world MARD was calculated to be 18.62 ± 11.87%. This value is higher than MARDs reported in recent literature concerning other CGM systems in non-critical hospitalized patients, such as 12.1% for the Dexcom G6 [7] and 12.7% in a large observational study [8]. Even studies focused on critical patient settings have reported lower values (e.g., 14.6% in critical and non-critical care [5] and 14.7% in post-cardiac surgery critical care [10]). This elevated MARD suggests that the physiological complexity and volatility inherent to the IMICU environment may compromise FreeStyle Libre 2 sensor performance compared to lower-acuity settings.

Despite this elevated MARD, the clinical safety profile was robust. Specifically, 99.03% of all paired readings fell within the clinically acceptable Zones A (54.59%) and B (44.44%) in the Parkes EGA. The clinical safety is further corroborated by the point accuracy metrics, which show how performance improves when considering wider, yet still clinically actionable, tolerance bands. At the rigorous 15%/15 mg/dL threshold, only 46.14% of pairs were accurate. However, as the tolerance is relaxed, accuracy increases significantly: 61.11% of pairs were accurate within 20%/20 mg/dL, and a substantial 85.75% of pairs were accurate within 30%/30 mg/dL. For instance, the Finn et al.study in critical and non-critical care reported 84.8% of readings within 30%/30 mg/dL [5], a finding comparable to our own. This indicates that while FreeStyle Libre 2 may not meet the highest precision standards of outpatient use, its high accuracy at the 30% level, combined with the minimal (0.24%) presence in Zone E, strongly suggests that the device is reliable for detecting clinically significant hypo- and hyperglycemia. This divergence, high MARD but high Zone A+B percentage, supports the consensus view that while FreeStyle Libre 2 may not replace POC testing for insulin dosing due to variable accuracy, its ability to keep 99% of readings in safe zones makes it a powerful adjunctive tool for trend surveillance, providing valuable trend data and safety alerts while relying on confirmatory checks for definitive insulin dosing [11].

A significant proportion of our cohort (9 (39.13%)) had no history of diabetes but required monitoring due to factors like glucocorticoid therapy and enteric/parenteral nutrition. The literature supports this trend, highlighting the expansion of CGM applications to manage non-diabetic conditions such as steroid-induced hyperglycemia and PN-related glucose management [9]. This growing utility led Patham et al. to propose that real-time CGM should be considered an essential monitoring modality in the intensive care setting, specifically to capture stress-induced hyperglycemia, which is strongly associated with adverse outcomes and mortality [15]. Our study demonstrated that FreeStyle Libre 2 sensor performance was consistent regardless of diabetes status, validating the feasibility of using this technology reliably to monitor glucose volatility induced by therapeutic interventions in the high-acuity, non-diabetic population.

A notable and counterintuitive finding of this study was that sensor accuracy was better (lower MARD) in patients presenting with shock and acute kidney injury compared to those without these conditions. However, these results are exploratory and hypothesis-generating, particularly given the extremely small sample size in the shock (n = 2) and acute kidney injury (n = 7) subgroups. Given that hypoperfusion and severe illness are typically associated with degraded interstitial sensor function [5,8,10], this result warrants cautious interpretation. This finding is in contrast to documented evidence that accuracy may degrade in patients with unstable conditions like severe edema or those on vasopressors [5,8]. Crucially, we observed that patients receiving intravenous fluid therapy demonstrated significantly lower MARD. We hypothesize that fluid administration could improve tissue perfusion and the equilibrium between interstitial and blood glucose. This could also partially explain the results found in the shock group, as these patients typically receive aggressive fluid therapy. Additionally, patients with acute kidney injury also exhibited a statistically lower MARD. While statistically significant, the extremely small sample size in the shock (n = 2) and AKI groups (n = 7) makes these findings difficult to generalize, and it likely does not reflect typical sensor performance in hypoperfused states. In contrast, the MARD was not significantly affected by other common clinical factors, including age, BMI, and overall glycemic range (hypoglycemia vs. hyperglycemia). This aligns with previous reports that have also noted consistent performance across different patient demographics, suggesting that MARD variability in hospitalized patients may be more strongly tied to acute illness severity and therapeutic management than chronic patient factors [16].

The strengths and limitations of this study should be considered when interpreting the results. A primary strength is the unique setting, the IMICU, which addresses a critical gap in the literature by validating CGM performance in a Level 2 critical care environment. Furthermore, the inclusion of a substantial proportion of patients without a history of diabetes, monitored for hyperglycemia induced by medications or nutrition, is a key strength that supports the expanded use of CGM in non-traditional patient cohorts. The prospective, observational design further enhances the generalizability as it reflects the device's use in routine clinical practice. Furthermore, the inclusion of a detailed subgroup analysis contributes novel, though preliminary, data on performance during acute physiological instability. 

However, several limitations must be acknowledged. First, the study’s single-center location and small sample size (n = 23), particularly within critical subgroups, limit the statistical power and generalizability of certain findings. Second, paired glucose measurements were treated as independent observations, despite repeated measures per patient, which violates the assumption of statistical independence and may lead to underestimation of variance and overstatement of statistical significance. Third, we did not apply retrospective time-lag correction to the sensor data, as was done in some clinical trials [16]. While such corrections allow for the determination of 'true' sensor variability by accounting for physiological delay, we aimed to assess the system's performance in a real-world clinical workflow where such corrections are unavailable to the treating team, an approach consistent with recent observational studies [5,8]. Consequently, our MARD includes deviations caused by physiological lag, particularly during the rapid glycemic shifts common in intermediate care.

## Conclusions

This observational study supports the clinical viability of the FreeStyle Libre 2 system as an adjunctive monitoring tool in the high-acuity IMICU setting. It should not be used for insulin dosing decisions, consistent with current guidelines. Despite a higher numerical MARD than controlled studies, the system demonstrated a robust safety profile and consistent performance across diverse patient cohorts, including non-diabetic patients requiring glucose management for therapeutic interventions. Future studies should focus on optimizing sensor protocols and validating subgroup findings in larger, multicenter trials.
